# Supplementary material for: Epidemiological Study of Malignant Paediatric Liver Tumours in Denmark 1985–2020
Source: Cancers (Basel). 2023 Jun 26;15(13):3355. doi: 10.3390/cancers15133355 (PMC10341131; doi:10.3390/cancers15133355)
Supplement: Supplementary file 1 [file cancers-15-03355-s001.zip › cancers-2440335-Supplement.pdf]

cancers

MDPI

Epidemiological study of malignant paediatric liver tumors in Denmark 1985-2020

Thomas N. Nissen, Catherine Rechnitzer, Birgitte K. Albertsen, Lotte Borgwardt, Vibeke B. Christensen, Eva Fallentin, Henrik Hasle, Lars S. Johansen, Lisa L. Maroun, Karin B. Nissen, Allan Rasmussen, Mathias Rathe, Steen Rosthøj, Nicolai A. Schultz, Peder S. Wehner, Marianne H. Jørgensen and Jesper Brok

Table S1. Characteristics of study participants with Hepatocellular Carcinoma or other liver tumors.

	HCC N = 9	Other N = 9
	**n (%)**	**n (%)**
Sex (male)	2 (22)	6 (67)
Age at diagnosis (years, median IQR)	9.64 (6.92-13.76)	1.96 (1.40-10.05)
Alpha fetoprotein-1 ug/L (median, IQR)	86,338 (4,867-2,218,000)	1.5 (1-2)
Extrahepatic vessels	2 (22)	0 (0)
Local nodes	1 (11)	1 (11)
Distant metastases	2 (22)	4 (44)
Pre surgery chemotherapy		
Cisplatin	0 (0)	0 (0)
Cisplatin/Doxorubicin	1 (11)	0 (0)
Cisplatin/Doxorubicin/Carboplatin	2 (22)	1 (11)
NA	6 (67)	8 (89)
LTX	5 (56)	0 (0)
RescueLTX	0 (0)	1 (11)
Type of primary surgery		
LTX	5 (56)	0 (0)
Minor liver resection (<3 segments)	0 (0)	1 (11)
Hemi hepatectomy	1 (11)	3 (33)
Extended hemi hepatectomy	0 (0)	1 (11)
Inoperaple	2 (22)	3 (33)
No tumor	0 (0)	1 (11)
Post-surgery chemotherapy		
Cisplatin	0 (0)	0 (0)
Cisplatin/Doxorubicin	0(0)	0 (0)
Cisplatin/Doxorubicin/Carboplatin	1 (11)	0 (0)
None	0 (0)	1 (11)
NA	8 (89)	8 (89)
Relapse	1 (11)	2 (22)

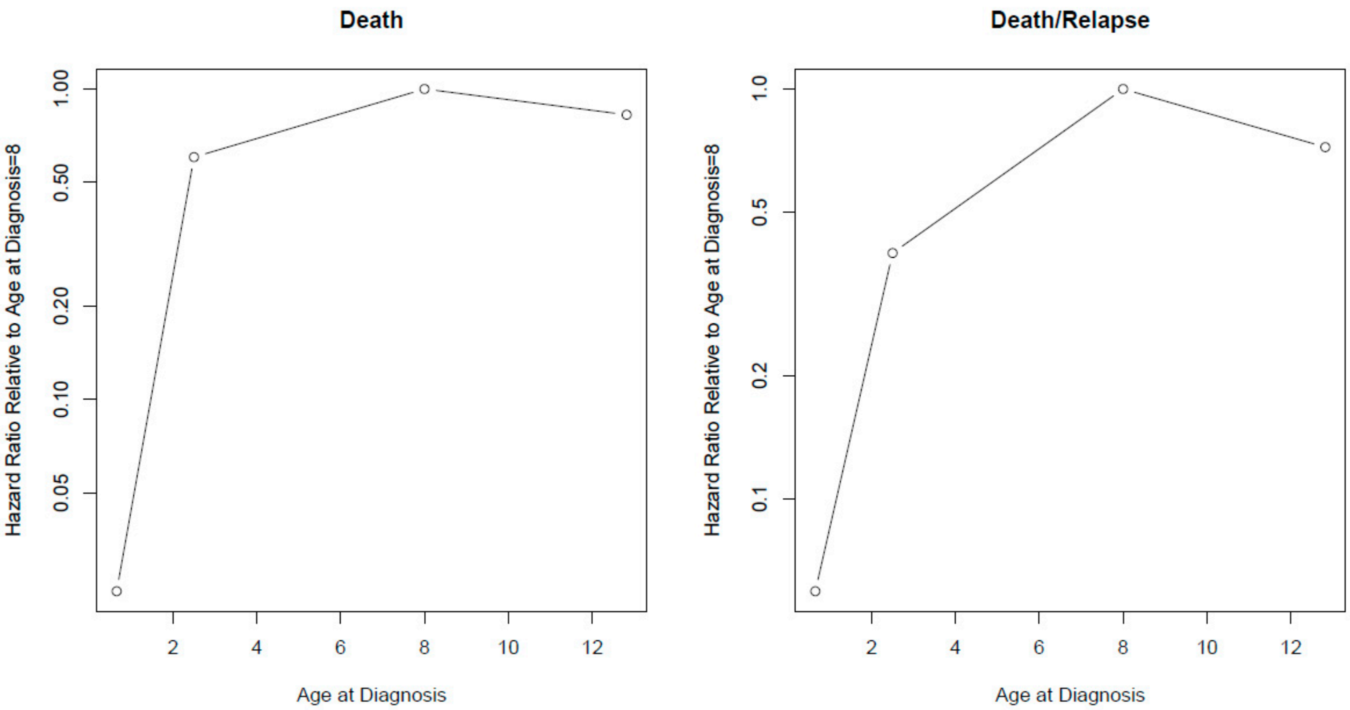

Figure S1. Rplot spline 2.5 and 8 yr.

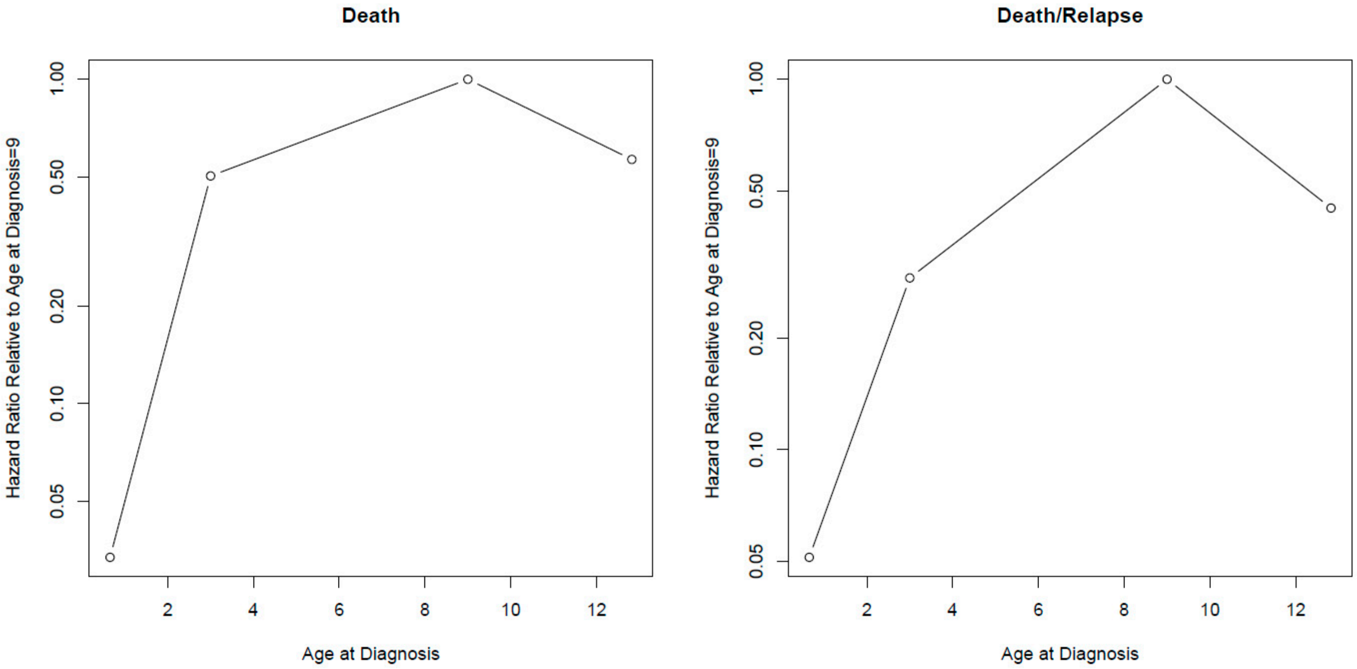

Figure S2. Rplot spline 3 and 9 yr.

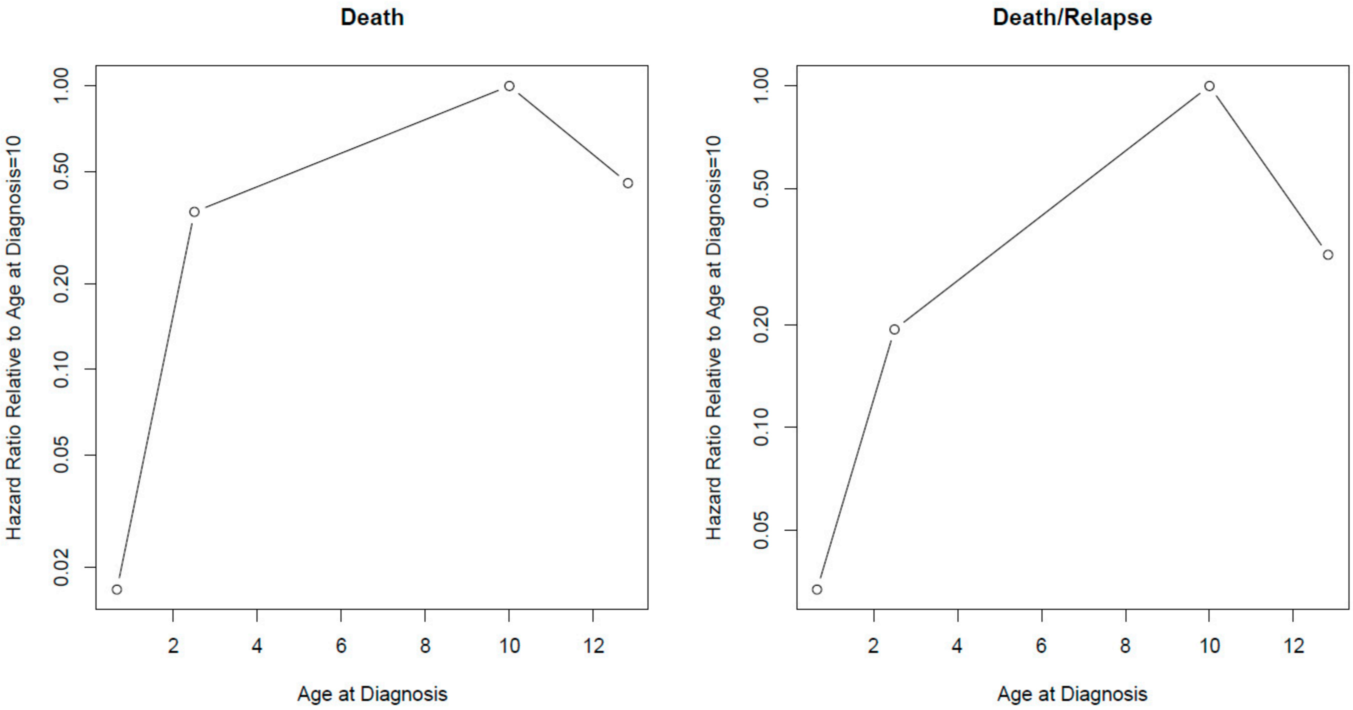

Figure S3. Rplot spline age 2.5 and 10 yr.

Disclaimer/Publisher's Note: The statements, opinions and data contained in all publications are solely those of the individual author(s) and contributor(s) and not of MDPI and/or the editor(s). MDPI and/or the editor(s) disclaim responsibility for any injury to people or property resulting from any ideas, methods, instructions or products referred to in the content.
